# THE RELATIONSHIP BETWEEN HAIR CORTISOL, CHRONIC STRESS, AND WELL-BEING AMONG OLDER ADULTS WITH DEMENTIA

**DOI:** 10.1093/geroni/igz038.1747

**Published:** 2019-11-08

**Authors:** Eunsaem Kim, Cory Bolkan, Erica Crespi, Jennifer Madigan

**Affiliations:** 1 Washington State University, Vancouver, Washington, United States; 2 Washington State University, Pullman, Washington, United States

## Abstract

Hair cortisol concentration (HCC) is a new method of measuring chronic stress by analyzing a small sample of hair. Given the strong relationship between chronic stress and quality of life (QOL) in people with dementia, HCC may enhance our understanding of their well-being. Our primary goal was to establish feasibility of HCC testing in people with dementia as a biomarker of chronic stress. To do so, we examined the HCC levels of chronic stress during the transition to memory care. Newly admitted memory care residents (N = 13, mean age = 82) were followed over nine months. Residents’ hair samples and health information were collected at three-month intervals. HCC in individuals with dementia (16.29 pg/mg) were considerably lower than older adults without dementia (30.48-57.8 pg/mg). Transition to memory care was associated with significantly decreased HCC levels (β = -.70, p < .001). Depression (β = -.32, p < .05) and psychotropic medication use (β = -.20, p < .01) facilitated the rate of HCC decline. Additionally, greater fluctuations in HCC over time were associated with higher HCC levels (β = .048, p < .05). Elevated HCC levels were related to poor functional capabilities (β = 8.31, p < .05), low social engagement (β = -6.01, p < 05), and poor overall QOL (β = -3.61, p < .01). These results underscore the significance of positive care environments to minimize chronic stress in individuals with dementia and indicate that HCC may be a useful stress measure in this population.

